# Bidirectional knotless barbed versus conventional smooth suture for closure of surgical wounds in inguinal castration in horses

**DOI:** 10.1186/s12917-020-02449-6

**Published:** 2020-07-17

**Authors:** Ditte Marie Top Adler, Stine Østergaard, Elin Jørgensen, Stine Jacobsen

**Affiliations:** 1 Department of Veterinary Clinical Sciences, University of Copenhagen, Taastrup, Denmark; 2 Department of Pharmacy, University of Copenhagen, Copenhagen, Denmark

**Keywords:** Equine castration, Barbed suture, Surgery

## Abstract

**Background:**

Castration of the stallion is one of the most frequently performed surgical procedures in the horse. Recently barbed suture materials for surgical wound closure were introduced to the market with manufacturers claiming that these sutures enhance speed and security as they eliminate the need to tie knots. Recently, it has been suggested that this type of suture may increase postoperative complications. This study aimed at investigating and comparing a bidirectional absorbable knotless barbed suture (KBS) to a conventional smooth suture (SS) for wound closure of inguinal castrations in the horse. This was done by evaluating short-term and post-discharge complications and by comparing the time spent on suturing the surgical wounds after bilateral inguinal castration, which was performed on 45 horses undergoing castration at The Large Animal Teaching Hospital at University of Copenhagen from September 2017 to May 2019.

**Results:**

Short-term complications were few; at 24 h minor swelling occurred in 29 and 33% of horses sutured with KBS and SS respectively and cutaneous dehiscence during recovery occurred in two horses of each group. Post-discharge follow-up revealed that three horses needed veterinary attention for treatment of complications (scrotal swelling (*n* = 1, KBS); wound exudation (*n* = 1, SS) and wound dehiscence after return to pasture (*n* = 1, SS)). Wound closure was achieved 6 min faster with KBS than with SS (*P* < 0.0001).

**Conclusions:**

Use of the KBS suture did not result in increased occurrence of postoperative complications. Wound closure was faster with KBS than with SS in equine bilateral inguinal castration. Our results show that KBS can safely be used in the horse following bilateral inguinal castrations without adverse effects and with a reduction in suturing time.

## Background

In equine practice, castration of the stallion is a routine surgical procedure. A variety of different approaches exist and castration can be performed in both the standing and recumbent horse. In the recumbent horse, castration is performed by either a scrotal or an inguinal approach, and when castration is performed under general anaesthesia in hospital conditions and using strict surgical asepsis, wounds are sutured for primary healing [1, 2].

Recommended suture material for closure of the incision following castration is a size 2–0 monofilament absorbable suture [3]. Monofilament suture is generally recommended for skin closure in horses [4]. In more recent years, barbed suture materials has been introduced in both human and veterinary medicine for closure of a variety of different surgical wounds [5–11]. Manufacturer claims that this type of suture enhances speed and security as it eliminates the need to tie knots.^1^ Nonetheless, this has not been investigated in the horse for skin closure in elective procedures such as castrations.

It has been suggested that the design of barbed sutures make them prone to postoperative infection [12]. In particular, accumulation of bacteria on the increased surface area under the barbs [12] and the ability of the barbs to trap fibres from towels or gauze [12, 13], have been suggested to increase postoperative wound infection. However, this has not yet been assessed in horses.

The primary objective of the study was to evaluate postoperative complications of wounds sutured with KBS and SS with special emphasis on surgical site infections (SSIs) and suture-holding capacity. The secondary objective was to investigate suturing time for closure of surgical wounds after bilateral inguinal castrations using either a bidirectional absorbable knotless barbed suture (KBS) or a conventional smooth suture (SS).

We hypothesized that the incidence of adverse effects would be similarly low in wounds sutured with two types of suture material and that closure of the surgical wounds would be achieved faster with KBS than with SS.

## Results

### Horses

A total of 45 horses underwent bilateral inguinal castration. Twenty-four were sutured with SS and 21 with KBS. Breeds included Icelandic horse (*n* = 14), Warmblood (*n* = 14), Friesian horse (*n* = 2), Pura Raza Española (*n* = 2), Welch Cob (*n* = 3), Holsteiner (*n* = 1), Irish Cob (*n* = 1), Oldenburg (*n* = 1), ox Arabian Horse (*n* = 1), Palomino (*n* = 1), Thoroughbred (*n* = 1), Shetland Pony (*n* = 1), Irish Sport Horse (*n* = 1), Pinto (*n* = 1), mixed breed (*n* = 1). The mean age of the SS group was 3 years and 10 months (range, 2 months–7 years). The mean age of the KBS group was 4 years and 7 months (range, 1 year 3 months–12 years). The mean weight of the SS group was 381 kg (range, 128–545 kg). The mean weight of the KBS group was 429 kg (range, 267–590 kg). There were no differences in weight or age (*P >* 0.05) between the two groups. Twelve of 45 horses were uni- (*n* = 11) or bilaterally (*n* = 1) inguinal cryptorchid. Eight of those were sutured with SS and four were sutured with KBS.

### Prevalence of complications and statistical analysis

The difference in proportions of complications between the six surgeons was not statistically different (*P* = 0.87). Therefore, the data for complications were pooled for further analysis. For the pooled analysis, there was no statistically significant difference in short-term or post-discharge complications between horses sutured with KBS and with SS (*P* > 0.05).

### Short-term complications

All (*n* = 45) horses were hospitalized for 24 h post-surgery. Thirteen horses were discharged after 24 h, leaving 32 horses (18 sutured with SS and 14 sutured with KBS) to be monitored for 48 h after surgery. After 48 h, an additional 19 horses were discharged, leaving 13 horses to be monitored for 72 h post-surgery (eight horses sutured with SS and five sutured with KBS).

**Fig. 1:**
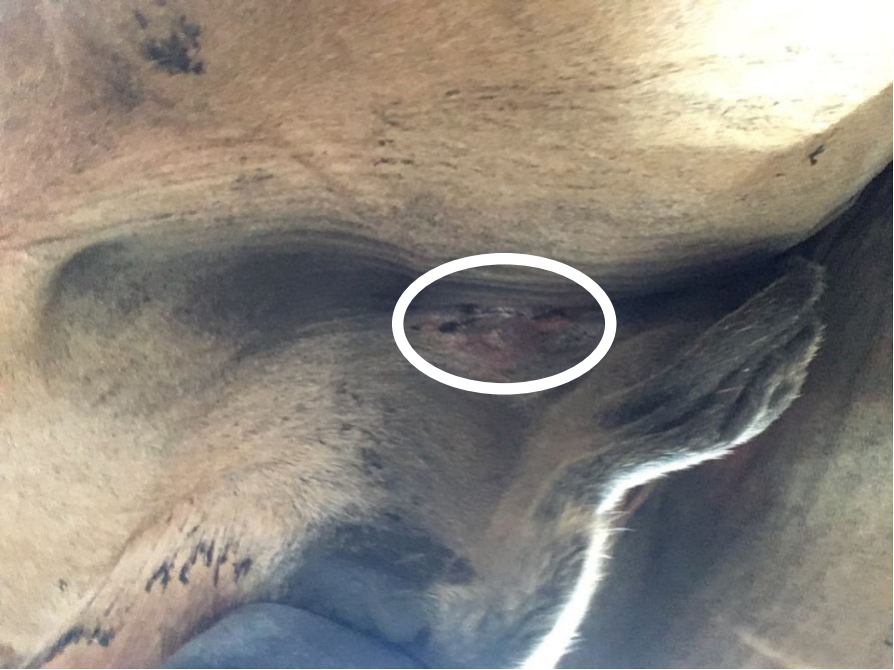
Surgical wound with no swelling

**Fig. 2:**
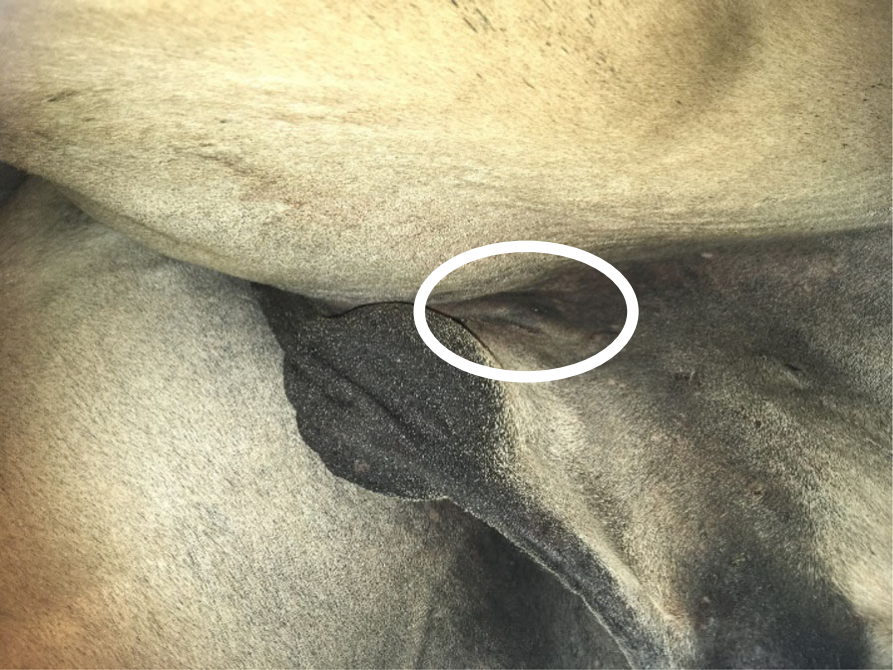
Surgical wound with minor swelling

At 24 h post-surgery, 6/21 (29%) horses sutured with KBS had a minor swelling in relation to the surgical wound uni- or bilaterally and 8/24 (33%) horses sutured with SS had a similar minor swelling uni- or bilaterally (Figs. 1 and 2). Two horses sutured with SS and two sutured with KBS had minor or moderate cutaneous dehiscence, which developed during recovery. It was the same surgeon (surgeon E), who performed the two castrations, where dehiscence occurred in the SS group and the same surgeon (surgeon D), who performed the two castrations, where dehiscence occurred in the KBS group. At 48 h post-surgery 7/14 (50%) horses in the KBS group and 6/18 (33%) in the SS group had a minor swelling uni- or bilaterally in relation to the surgical wound. At 72 h post-surgery, 4/5 (80%) horses in the KBS group and 3/8 (38%) in the SS group had a minor uni- or bilateral swelling in relation to their surgical wounds. Exudation was not observed in any horse.

### Post-discharge complications

Telephone interviews with owners were performed at a median (range) of 5 months (1.5–11 months) after castration. Despite efforts of repeated contact, 2/45 owners were still unreachable at 11 months post-surgery, and were not contacted further. Two owners contacted the hospital by themselves for unrelated matters 1.5 months post-surgery and were interviewed at that time point.

The owners of 1/24 horses sutured with SS reported that their horse had a minor swelling related to one of the surgical wounds 2 days after discharge, which resolved without intervention after a few days.

Three horses needed veterinary attention after discharge due to wound-related complications. One horse sutured with SS developed exudation from one of the surgical wounds 5 days post-surgery (which was 4 days after discharge from the hospital), but showed no other signs of infection. This was a 2-months old foal, with no previous signs of wound complication. A local veterinarian instituted treatment with sulfadiazine/trimethoprim for 8 days, during which exudation ceased. The second horse that needed veterinary attention was sutured with SS and had no complications within the first 3 weeks after surgery. However, after being turned out into pasture, it developed slight dehiscence in one wound accompanied by a swelling around the surgical area and fever. A local veterinarian instituted penicillin treatment for 5 days after which signs of infection ceased. The third horse, who needed veterinary attention after discharge was one (from the KBS group) of the four horses (two from each group) that developed cutaneous dehiscence during recovery. The horse needed veterinary attention after discharge due to swelling of the scrotum. Penicillin treatment for 5 days was initiated by the attending veterinarian, who also opened up the wounds, which at this time point had closed. This treatment was repeated after 5 days. The remainder three horses with post recovery cutaneous dehiscence developed minor exudation from the wounds postoperatively and scrotal swelling, which resolved without further treatment.

### Suturing time

Total suturing time was 15.1 min (SD = 4.0 min) for SS and 9.1 min (SD = 2.2 min) for KBS. Overall individual skin incisions ranged in length from 3.0–8.8 cm with no difference (*P* > 0.05) in length between wounds sutured with SS and KBS (average total incision size of KBS = 10.8 (SD = 2.6) and SS = 11.0 (SD = 2.0) respectively). Wound closure was achieved faster with KBS than with SS (*P* < 0.0001). Wound closure with KBS was 6 min faster than with SS, which corresponds to a 40% reduction in suturing time; all surgeons closed wounds faster with KBS than SS (Table 1).

**Table 1:** Suturing time in seconds/cm for six surgeons performing wound closure after inguinal castration using knotless barbed (KBS) or smooth (SS) suture material

Surgeon	n _KBS_	n _SS_	Wound closure time in sec/cm (± SD)		
			KBS	SS	Difference (SS-KBS)
A	9	9	47.60 (12.65)	89.91 (24.31)	42.31
B	4	3	68.15 (18.30)	85.83 (20.24)	17.38
C	2	5	35.66 (3.25)	62.62 (4.80)	26.96
D	4	4	49.40 (5.85)	77.08 (6.38)	27.68
E	1	2	71.62	119.86 (17.75)	48.24
F	1	1	50.00	63.16	13.16
Total	21	24			

## Discussion

Wound closure with the KBS did not result in short-term or post-discharge complications exceeding those found after wound closure using SS (*P* > 0.05). The KBS resulted in faster wound closure than with the conventional SS material (*P* < 0.0001) for all surgeons (Table 1). In equine surgery, KBS has been used experimentally for laparoscopic cystorrhaphy [14, 15], jejunal end-to-end anastomoses [16], caecal and pelvic flexure enterotomies [17, 18] and for ablation of the nephrosplenic space [8]. Three previous studies have reported the use of barbed suture in equine clinical cases for ablation of the nephrosplenic space in eight horses [19], for uteropexy in three mares [9], and for closure of the vaginal rings in a horse [6]. In all three clinical studies, the surgical procedures were performed laparoscopically, and authors found that the barbed sutures facilitated the procedures by obviating need for intracorporeal knot tying.

While many studies have investigated use of barbed sutures for laparoscopic procedures [6, 9, 19], suturing viscus [5, 7, 11, 16–21] or the deep layers in arthrotomy [12, 22, 23], fewer have assessed KBS in dermal, subcutaneous, and subcuticular closure. Some studies on knee and hip arthroplasty in humans found increased complication rates (infection, dehiscence) when KBS was used to close superficial tissue layers [12, 23, 24]. A recent metaanalysis including data from nine studies in humans did, however, conclude that closure of arthrotomies and subcutaneous and subcuticular tissues with KBS appeared to decrease the total complication rate in knee arthroplasty compared to suturing with traditional knotted sutures [25].

None of the horses in our study developed SSIs during hospitalisation (up to 72 h). Absence of SSIs in the immediate postoperative period are in line with previous studies reporting complications after castration using the inguinal approach [1, 26]. No horse developed fever in the immediate postoperative period in our study. This is in contrast to previous studies reporting fever in 2.5–21.4% (4/159 and 51/238) of horses [1, 26]. The reason for this difference is not entirely clear, but it may be related to differences in pre-, intra- and postoperative treatments between studies. A limitation to our study is the fairy low number of horses included, which might result in non-detection of very rare complications.

In our study, short-term complications after suturing with both KBS and SS were mild in all horses. Swelling of the suture line was absent or minor in all horses, and none of the horses had exudation from their wounds. These similar findings after suturing with KBS and SS are comparable to previous studies in man [11, 27] where use of KBS in laparoscopic myomectomies [11] and non-emergent caesarean section [27] resulted in wound healing similar to that found after suturing with SS. Short-term complications observed in the present study were for all cases deemed to be within normal limits, and no additional treatments were instituted nor was discharge of horses delayed. Four horses (two SS and two KBS) developed unilateral wound dehiscence during recovery. Wound dehiscence after castrations with primary closure have previously been reported [1]. While such complications could be related to the suturing and materials used, we feel that they are most likely associated with either the stress put on the wounds from recovery or from individual surgeons’ technique, as it was the same surgeon (surgeon E), who performed the two castrations, where dehiscence occurred in the SS group and the same surgeon (surgeon D), who performed the two castrations, where dehiscence occurred in the KBS group.

The frequency of observed short-term complications were generally comparable to those observed in previous studies [1, 26]. Our most frequent short-term complication was minor swelling at the suture line. These findings are comparable to findings by Kummer et al. [1], who described short-term complications after bilateral inguinal castrations using a similar SS suture. Kummer et al. [1] reported mild swelling at the surgery site to occur in 37.4% of cases compared with 28 and 33% in the 2 groups of horses in our study. Kummer et al. [1] also reported occurrences of moderate swelling (5%) and severe swelling (0.4%), which we did not observe in our study. Scoring of swelling was performed subjectively, which hampers direct comparison between studies.

There was no difference (*P* > 0.05) in post-discharge complications between horses sutured with KBS and those sutured with SS. These findings are in line with long-term findings in recent human studies comparing dermal closure with barbed and traditional sutures [28]. Previously, a study comparing smooth and barbed suture for dermal closure in cosmetic surgeries (abdominoplasties, mastopexies, and reduction mammoplasties) in man also found comparable complication profiles for the two suture types at 3-month follow-up [29]. Post-discharge, three horses needed veterinary attention due to wound-related complications and the attending veterinarians instituted antibiotic treatment in those three horses. One of the three horses treated with antibiotics post-discharge was one of the four horses who had partial wound dehiscence during recovery from surgery, whereas the remainder two did not previously present with any wound related complications.

Faster closure using KBS was previously demonstrated in gynaecologic and urologic surgery in man [11, 20], in jejunal anastomosis construction in horses (ex vivo) [16] and in closure of jejunal enterotomies in dogs (in vivo) [7]. In the present study, colour, material, and needles of the two sutures were kept as similar as possible, and the longer closure time using SS may thus most likely be attributed to the time spent on knot tying. However, other factors related to the design of the KBS may have affected closure time. In particular, the barbs of the suture prevents the suture from loosening and backsliding during closure, potentially reducing the time spent on tissue approximation during suturing. The fact that the suture was bidirectional rather than with a loop may have reduced suturing time even further, as this design eliminates the time spent on anchoring the loop end at the beginning of the suture line. The bidirectional design further makes it suitable for closing two different tissue layers (one end for the fascia/subcutis and one end for the dermis). To investigate further if the time saved by closure of wounds with KBS compared with SS was attributed the knot tying procedure itself, measuring time spent specifically on knot tying with SS could have been interesting. Other suture-related factors (e.g. if the suture is easier to handle or does not backslide) may add to the faster suturing, but differentiating these potential causes of faster suturing is not possible from our study.

Generally, the suture costs are higher for barbed sutures compared with conventional sutures. In the present study, costs for the KBS were four times that of the SS (13.6 € and 3.4 € excluding VAT, respectively). Use of KBS reduced suturing time with approximately 6 min per case corresponding to a 40% reduction in suturing time. The added cost of the KBS suture must thus be weighed against savings related to reduced operating room time (personnel, anaesthesia etc.) and patient gains from a reduced anaesthesia time. For elective surgical procedures in healthy animals, the few minutes of anaesthesia is not likely to influence outcome significantly, but a 40% decrease in suturing time may be crucial in the critically ill patient or during closure of longer wounds such as for instance closure of the subcutaneous tissue in colic surgery.

## Conclusions

In conclusion, our study demonstrated that closure of surgical wounds after bilateral inguinal castrations in horses with KBS did not increase occurrence of postoperative complications after castration. Our results thus suggest that the bidirectional KBS can be used safely for closure of subcutaneous and dermal tissues in inguinal castrations in the horse. Further, use of KBS was faster with the bidirectional KBS than with the SS counterpart.

## Methods

### Study design

Forty-five normal and inguinal cryptorchid stallions admitted for castration to The Large Animal Teaching Hospital, University of Copenhagen from September 2017 to May 2019 were included in the study. Horses were included in the study if they had normal descended testicles present in scrotum or if they were uni- or bilateral inguinal cryptorchids. Stallions were excluded from the study if they were abdominal cryptorchids or if they were to have more surgical procedures performed during the same anaesthetic period (e.g. arthroscopic fragment removal).

All horses were castrated using the same technique and the surgical wounds were sutured for primary closure. Horses were randomly assigned to be sutured with either a size 2–0 smooth, synthetic, absorbable monofilament suture of glycolide and e-caprolactone (Monocryl, Ethicon, Somerville, NJ) (SS) or a size 0 (corresponding to tensile strength 2–0)^2^ bidirectional barbed, synthetic, absorbable, monofilament suture of glycolide and e-caprolactone (Quill, Surgical Specialties Corporation, Westwood, MA) (KBS).

In the selection of the SS suture for comparison with KBS, it was not possible to identify a complete match concerning needle profile. As Surgical Specialties Corporation specializes in knotless tissue closure devices,^3^ the company does not market smooth counterparts to their knotless sutures, but lists Monocryl as a comparable suture.^4^ Needles of both sutures were swaged, curved, and reverse cutting. It was not possible to find a 2–0 Monocryl suture with a needle of exactly the same length and curvature as the Quill suture, and the suture with the closest needle match was therefore chosen: a 24 mm, 3/8, reverse cutting needle (Monocryl) compared with a 26 mm, 1/2-circle, reverse cutting needle (Quill). The selected sutures were of similar material (both copolymers of glycolide and caprolactone) and colour (both undyed), but differed in size. The difference in suture size was deliberate. Size of sutures is determined by outer diameter of the suture. Barbs of KBS are created by cutting into the core of the suture, which effectively reduces the diameter of the suture [30]. While other producers of unidirectional KBS (e.g. V-Loc, Medtronic, Minneapolis, MN) [31], have taken this into account and labeled their sutures accordingly (so that users of KBS select a suture size analogous to the size that would be selected if standard smooth suture material was used), this is not the case for Quill sutures. We thus upsized the KBS by one as recommended by the manufacturer^5^ to effectively have similar size KBS and SS.

The barbed suture design eliminates the need to tie knots and further improve tissue apposition by a more even distribution of tension on the soft tissue [29, 30]. The barbs of the bidirectional suture is set in opposing directions from either side of the suture midpoint,^6^ allowing the use of the suture in two directions. The bidirectional suture therefore has a needle attached to both ends of the suture. Unidirectional sutures, such as for instance V-Loc (Medtronic), has a pre-constructed loop end, which is used to anchor the suture in the tissue at the beginning of a suture line. This structure allows passage of the suture through tissue in a single direction only. In our study the bidirectional KBS suture was used to close the subcutaneous tissue with one end of the suture, whereas the other end of the bidirectional KBS was used to close the skin.

The study was approved by the Ethical and Administrative Committee of the Department of Veterinary Clinical Sciences, University of Copenhagen (reference number: 2017–002) and horse-owners of enrolled stallions signed an informed consent form.

### Anaesthesia and perioperative treatment

Horses were sedated with 0.01 mg/kg detomidine intravenously (IV) (Orion Pharma, Espoo, Finland), 0.03 mg/kg acepromazine IV (Pharmaxim, Helsingborg, Sweden); within 5 min, sedation was supplemented with 0.03 mg/kg butorphanol IV (Merck, Kenilworth, NJ, USA). Anaesthesia was induced with 1.5 mg/kg zolazepam and tiletamin IV (Virbac, Carros, France). Anaesthesia was maintained with isoflurane (Virbac, Carros, France) in oxygen using intermittent positive pressure ventilation.

Immediately prior to induction of anaesthesia, horses received one dose of 22.000 IU/kg benzyl penicillin IV (Panpharma S.A., Luitré, France) and a dose of 1.1 mg/kg flunixin meglumine IV (Merck, Kenilworth, NJ, USA). During hospitalization horses were treated IV with 1.1 mg/kg flunixin meglumine 24 and 48 h after surgery and 1.1 mg/kg PO once after discharge at 48 h if discharge occurred before 48 h after surgery. Horses which were not vaccinated with tetanus routinely, were administered tetanus antitoxin (Colorado Serum Company, Inc., Denver, USA) subcutaneously and anti-*Clostridium tetani* toxoid (Pfizer, NY, USA) intramuscularly. Horses were confined to a box stall for 24 h post-surgery and then walked by hand two-three times daily for 15 min. Horses were discharged 24–72 h post-surgery depending on owners’ wishes. Owners were instructed to keep horses stall rested and hand walk them until 8 days post-surgery; hereafter horses were turned out in a small paddock for two additional weeks after which there were no restrictions in regard to turn out or ridden exercise. Owners were further instructed to daily look underneath their horse and monitor the surgical wounds and scrotal area for swelling, discharge from surgical wounds, and call the hospital /a veterinarian in case of presence of any of the above.

### Surgical approach

Horses were positioned in dorsal recumbency. The scrotal and inguinal area were aseptically prepared for surgery and draped. Ten mL of 2% lidocaine (AstraZeneca, Cambridge, UK) was injected in each testicle when possible, i.e. when the testicle was present in normal scrotal position and/or when it was easily identified in the inguinal canal. Castration was performed by the inguinal approach by exposing the superficial inguinal ring through a skin incision centred over the superficial inguinal ring. Castration was performed by the closed technique. After separation of the parietal tunic surrounding the testis from the scrotal ligament and fascia by digital dissection, the testis and parietal tunic were separated from surrounding fascia using a gauze swap while placing mild traction on the testicle and cord. A Sand’s non-cutting emasculator was applied to the cord for 10–30 s with the purpose of crushing it briefly at the area of subsequent ligature placement. One transfixating, circumferential ligature of size 2 polyfilament braided lactomer (Medtronic, Minnesota, USA) was applied in the crushing groove created by the Sand’s emasculator and a second ligature was applied 2–3 cm proximally before the cord was severed distal to the ligatures. For security in case a ligature should slip we standardly double ligate the cord during castration. The subcutaneous tissue, fascia and skin were closed in two layers as recommended [3] using either SS or KBS. The fascia beneath the skin and the subcutaneous tissue were closed in one layer and the skin was closed in another layer. Both sutures were applied in a simple continuous pattern in the deep layer, while the skin was closed intradermally. For the SS closure, 4 surgeon’s knots were used per incision with a minimum of 4 throws per knot. For the KBS the anchoring of the suture at the end of the suture line was performed by adding two additional stitches to the subcutaneous line and by backstitching in the dermal layer. Six different surgeons carried out the castrations.

### Intraoperative variables and assessment of complications

Recorded variables were: 1) Length of the incision in cm as measured by a sterile ruler, 2) Closure time (seconds) measured from initial needle insertion until suture ends were cut off after dermal closure. Closure time was recorded for each side (right and left) and the average of the total time (right + left) spent on suturing each horse was used for statistical analyses. 3) Postoperative complications. Short-term complications were monitored daily during hospitalization until discharge or 72 h post-surgery, whichever occurred first. Clinical parameters (swelling, exudation, and dehiscence) were graded subjectively (none, minor, moderate or severe) based on presence in relation to the suture line and the surroundings of the suture line.

Post-discharge complications were assessed based on telephone interviews with the owners 2 months post-surgery. Those owners not available for contact at 2 months post-surgery were called repeatedly until contact or until 11 months after surgery, resulting in interviews performed between 2 and 11 months post-surgery. Owners were interviewed based on the discharge instructions (to daily look underneath their horse and monitor the surgical wounds and the scrotal area for swelling and/or discharge from the surgical wounds). Specifically horse-owners were asked if their horse had developed complications since discharge from the hospital, and where complications were present, owners were asked to describe them and report whether it had been necessary to request veterinary assistance to treat the complication(s).

The complications reported in the study are those related to SSIs and the suture line. Other short-term and post-discharge complications are reported in an additional file (see Additional file 1).

### Statistical analyses

Differences in proportions of the categorical variables of suture line complications between 1) SS and KBS and 2) the six surgeons performing the castrations were compared using chi-square or Fisher’s exact test, as appropriate. The quantitative variables of time, age, and weight were compared between the two suture groups using two-sided student’s t-test (after test for variance equality). Significance threshold was set at *P* < 0.05. Data was analysed using SAS/STAT® software. The quantitative variables were all normally distributed, assessed by the Shapiro-Wilk normality test.

## Supplementary information

**Additional file 1.** Other complications than suture line and SSI related complications. This file describes short-term and post-discharge complications other than those related to SSIs and the suture line experienced in the present study.

## Data Availability

The datasets used and/or analysed during the current study are available from the corresponding author on reasonable request.

## Abbreviations

KBS: Knotless barbed sutures
SSIs: Surgical site infections
SS: Smooth suture

## Footnotes

1 see: https://www.surgicalspecialties.com/suture-wound-closure-brands/quill-barbed-sutures/ (Link tested 8 November 2019)

2 See: https://www.surgicalspecialties.com/wp-content/uploads/2019/06/SSC-021-R8-Quill-CatalogFinal-low-res-1.pdf (Link tested 8 November 2019)

3 See: http://www.quilldevice.com/about (Link tested 8 November 2019)

4 See: http://www.quilldevice.com/material-guide-hp (Link tested 8 November 2019)

5 See: https://www.surgicalspecialties.com/wp-content/uploads/2019/06/SSC-021-R8-Quill-CatalogFinal-low-res-1.pdf (Link tested 8 November 2019)

6 See: http://www.quilldevice.com/about (Link tested 8 November 2019)
