# Supplementary material for: Bidirectional knotless barbed versus conventional smooth suture for closure of surgical wounds in inguinal castration in horses
Source: BMC Vet Res. 2020 Jul 17;16:250. doi: 10.1186/s12917-020-02449-6 (PMC7368766; doi:10.1186/s12917-020-02449-6)
Supplement: Supplementary file 1 — Additional file 1. Other complications than suture line and SSI related complications. This file describes short-term and post-discharge complications other than those related to SSIs and the suture line experienced in the present study. [file 12917_2020_2449_MOESM1_ESM.docx]

**Additional file 1**

Other complications than suture line and SSI related complications

During the first 24 h of surgery, two horses sutured with SS and one sutured with KBS developed a unilateral scrotal hematoma, two of which were drained through a scrotal incision 48 h post-surgery prior to discharge. No post-discharge complications in any of the horses.

Between 7-20 days of castration (post-discharge), three horses developed a non-infected scrotal seroma. One of which was drained through scrotal incisions due to its size (10 x 20 cm). No long-term complications in any of the horses.
